# Driving and Driven Architectures of Directed Small-World Human Brain Functional Networks

**DOI:** 10.1371/journal.pone.0023460

**Published:** 2011-08-12

**Authors:** Chaogan Yan, Yong He

**Affiliations:** State Key Laboratory of Cognitive Neuroscience and Learning, Beijing Normal University, Beijing, China; University of Michigan, United States of America

## Abstract

Recently, increasing attention has been focused on the investigation of the human brain connectome that describes the patterns of structural and functional connectivity networks of the human brain. Many studies of the human connectome have demonstrated that the brain network follows a small-world topology with an intrinsically cohesive modular structure and includes several network hubs in the medial parietal regions. However, most of these studies have only focused on undirected connections between regions in which the directions of information flow are not taken into account. How the brain regions causally influence each other and how the directed network of human brain is topologically organized remain largely unknown. Here, we applied linear multivariate Granger causality analysis (GCA) and graph theoretical approaches to a resting-state functional MRI dataset with a large cohort of young healthy participants (n = 86) to explore connectivity patterns of the population-based whole-brain functional directed network. This directed brain network exhibited prominent small-world properties, which obviously improved previous results of functional MRI studies showing weak small-world properties in the directed brain networks in terms of a kernel-based GCA and individual analysis. This brain network also showed significant modular structures associated with 5 well known subsystems: fronto-parietal, visual, paralimbic/limbic, subcortical and primary systems. Importantly, we identified several driving hubs predominantly located in the components of the attentional network (e.g., the inferior frontal gyrus, supplementary motor area, insula and fusiform gyrus) and several driven hubs predominantly located in the components of the default mode network (e.g., the precuneus, posterior cingulate gyrus, medial prefrontal cortex and inferior parietal lobule). Further split-half analyses indicated that our results were highly reproducible between two independent subgroups. The current study demonstrated the directions of spontaneous information flow and causal influences in the directed brain networks, thus providing new insights into our understanding of human brain functional connectome.

## Introduction

Recently, increasing attention has been focused on the investigation of the human brain connectome that describes the patterns of structural and functional connectivity networks of the human brain [1], [2]. Many studies have demonstrated that the human brain network follows a small-world topology (i.e., high clustering and short path lengths linking different nodes) [3]–[8] and has an intrinsically cohesive modular structure [9]–[11]. Importantly, these studies have also identified network hubs that are predominantly located in regions of the association cortices [3]–[7], [12], [13].

Despite the advances in research on the topological properties of human brain networks, most of these studies have focused on the undirected network analysis in which the directions of information flow and the neural driving architecture are overlooked. However, distinguishing the forward and backward connections and the construction of directed networks are important for describing the information interchange between brain regions and for better understanding the brain's function [14]–[16]. In clinical research, the study of directed network analyses with finding neural drivers is also essential for the identification of brain structures involved in the origin or the control of pathological activities, such as focal epilepsy [17]. Several studies in cats and monkeys have utilized anterograde and retrograde tracing techniques to investigate directed brain networks [18]–[20]. Although these techniques can be used to identify the information flow between brain regions, they cannot be applied to human beings in vivo because of their invasiveness.

Here, we used resting-state functional MRI (R-fMRI) data to investigate the driving and driven architecture of human brain directed network. R-fMRI is a powerful tool for the investigation of spontaneous neuronal activity of the human brain in health and disease because it has a lot of advantages such as reasonable spatial and temporal resolution, non-invasiveness and simplicity (participants don't need to perform specific experimental tasks) [21]–[23]. Recent advances in modern brain imaging techniques have suggested that R-fMRI allows for the mapping of the directed network of the human brain in vivo [24]–[27]. To construct the intrinsic whole-brain functional directed network, in this study, we utilized Granger causality analysis (GCA) [28] to obtain information flow directions between brain regions.

GCA incorporates information on temporal precedence and does not require *a priori* specification of a network model [29]–[31]. Therefore, this model is suitable for the construction of directed network of the human brain. Several recent studies have applied GCA to R-fMRI data to identify the information flow directions among a small number of regions [24]–[27]. Specifically, Liao et al. [27] utilized GCA on R-fMRI time series of 90 regions of interest to construct a directed whole-brain functional network at an individual level. However, they found that the small-world properties of the directed networks were very weak as the normalized clustering coefficients (the ratio of the clustering coefficient of the brain network to the constructed random networks) ranging from 1.02 to 1.08. This result was not compatible with previous undirected brain functional networks studies in which the normalized clustering coefficients were usually found to be around 2 [7], [8], [10], [32]. There are several possible reasons for the discrepancies. First, Liao et al. [27] used a kernel version of GCA that might over-fit the data and model too much noise. This processing could result in a very weak small-world property in the directed brain network under the given thresholds. The linear multivariate GCA can avoid the problems of fixing the degree of nonlinearity of the model and losing statistical power due to introducing more features with nonlinearity encountered in nonlinear generalization of GCA [33], [34]. Second, Liao et al. [27] constructed a directed network for each participant and analyzed the individual network properties. Notably, GCA may yield spurious connections (i.e., false positives) in the worst case scenario if the hemodynamic delay opposes the neuronal delay, and therefore, the causality needs to be statistically inferred [26]. Thus, in order to control the “false-positive” connections to be minimal (e.g., using conservative statistical criterion) in the brain network, it would be important to construct a population-based functional directed network by capturing the underlying common connectivity pattern of the brain.

To further clarify whether the directed brain functional network show small-world properties, in the present study, we used R-fMRI and linear multivariate GCA methods to construct a population-based directed network in the human brain. We further utilized graph theoretical approaches to analyze various topological properties of the brain networks, including the small-worldness, modules and hubs. Finally, we performed a split-half analysis to test the reproducibility of our results. We expected to discover prominent small-world characteristics and reliable driving and driven architectures in the directed human brain functional network.

## Materials and Methods

### Participants

Data were selected from a large sample R-fMRI dataset of our group, which has been publicly released as a part in the “1000 Functional Connectomes” Project (http://www.nitrc.org/projects/fcon_1000/). We selected 86 young healthy volunteers (48 females: 20.8±1.6 years old, range 18–25; and 38 males: 20.7±1.7 years old, range 17–25) with head motions of less than a 2.0-mm displacement in any of the x, y, or z directions or 2.0° of any angular motion throughout the resting-state scan and with a coverage of the whole brain as published previously [35], [36]. All participants were right-handed and had no history of neurological or psychiatric disorders. Written informed consent was obtained from each participant, and the study was approved by the Institutional Review Board of State Key Laboratory of Cognitive Neuroscience and Learning, Beijing Normal University.

### Image acquisition

MRI data were acquired using a SIEMENS TRIO 3-Tesla scanner in the Beijing Normal University Imaging Center for Brain Research. The participants were supine with the head snugly fixed by straps and foam pads to minimize head movement. During the resting-state session, the participants were instructed to hold still, keep their eyes closed but not fall asleep and not think of anything in particular. The functional images were obtained using an echo-planar imaging sequence with the following parameters: 33 axial slices, thickness/gap = 3/0.6 mm, in-plane resolution = 64×64, repeat time (TR) = 2000 ms, echo time (TE) = 30 ms, flip angle = 90°, field of view (FOV) = 200×200 mm. None of the subjects fell asleep according to a simple questionnaire after the scan. In addition, a T1-weighted sagittal three-dimensional magnetization-prepared rapid gradient echo (MPRAGE) sequence was acquired that covered the entire brain: 128 slices, TR = 2530 ms, TE = 3.39 ms, slice thickness = 1.33 mm, flip angle = 7°, inversion time = 1100 ms, FOV = 256×256 mm and in-plane resolution = 256×192.

### Preprocessing

Unless otherwise stated, all preprocessing was performed using Statistical Parametric Mapping (SPM5, http://www.fil.ion.ucl.ac.uk/spm) and Data Processing Assistant for Resting-State fMRI (DPARSF) [35]. The first 10 volumes of the functional images were discarded due to signal equilibrium and to allow the participants to adapt to the scanning noise. All slices of the remaining 230 volumes were corrected for the different acquisition times of the signals by shifting the signal measured in each slice relative to the acquisition of the slice acquired in the middle time of each TR. Then, the time series of images of each subject were motion-corrected using a least squares approach and a six-parameter (rigid body) linear transformation [37]. The individual structural image (T1-weighted MPRAGE images) was co-registered to the mean functional image after motion correction using a linear transformation [38]. The transformed structural images were then segmented into gray matter (GM), white matter and cerebrospinal fluid using a unified segmentation algorithm [39]. The motion corrected functional volumes were spatially normalized to the Montreal Neurological Institute (MNI) space and re-sampled to 3-mm isotropic voxels using the normalization parameters estimated during unified segmentation.

### GCA and network construction

**Figure 1:**
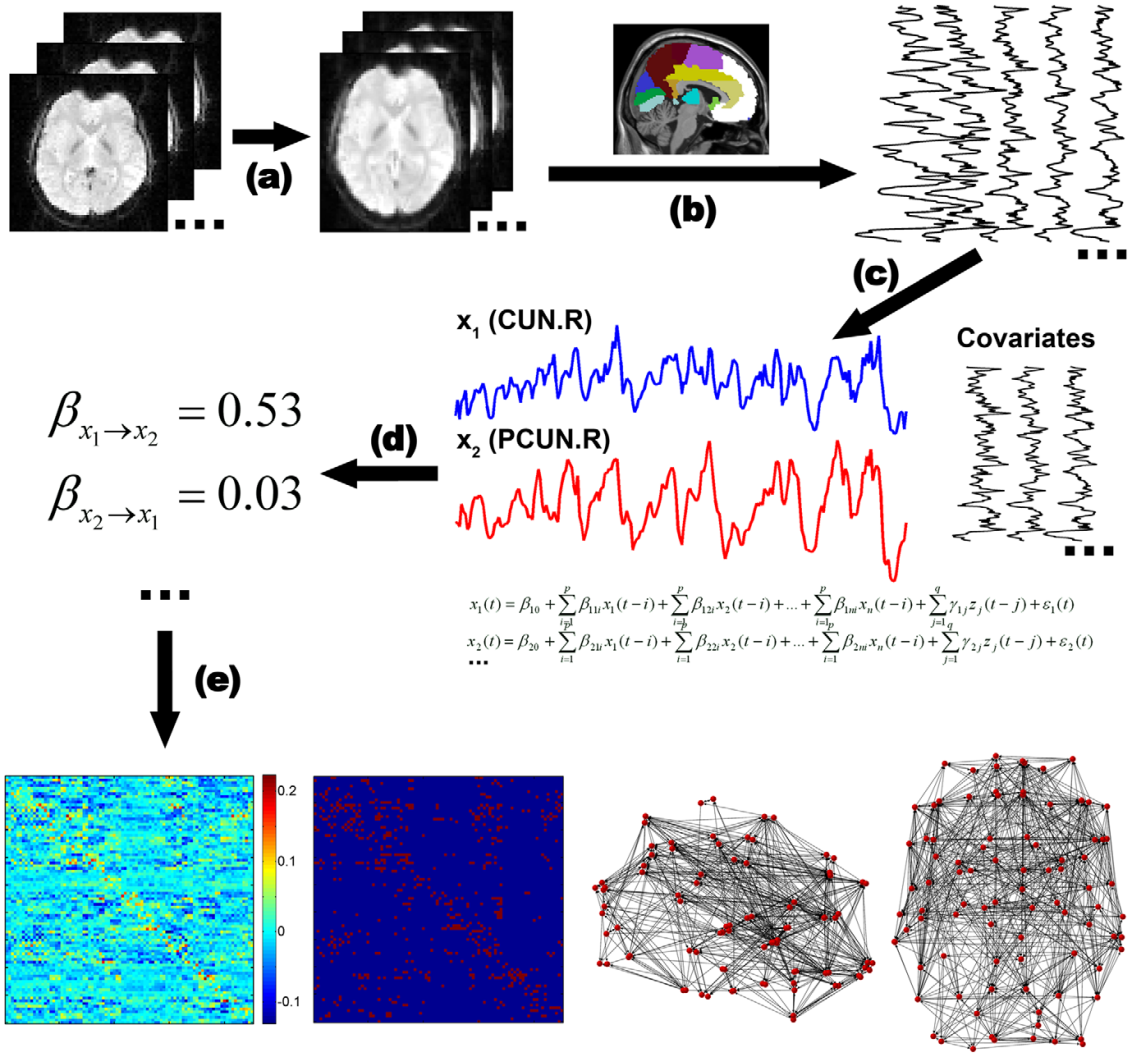
Flowchart for the construction of the human intrinsic whole brain functional directed network based on R-fMRI. (**a**) The resting-state functional images were preprocessed. (**b**) Mean functional time series of 90 AAL regions were extracted. (**c**) Multivariate GCA was applied to evaluate the relationship between the time series. (**d**) GCCs (β), which denote the causal influence between regions, were evaluated for each subject. (**e**) The functional directed network was constructed by exerting an FDR threshold on t-tests of the pair-wise GCCs across subjects. The left panel denotes the mean GCCs across subjects; the second left panel denotes the brain directed network after FDR correction; the right two panels denote the sagittal view and the axial view of the brain directed network.

The whole brain was first parcellated into 90 cortical and subcortical regions of interest (45 for each hemisphere, see Table S1) using a prior anatomical automatic labeling (AAL) atlas [40] (Figure 1). The mean time series of each region was extracted by averaging the time series of all voxels within that region. The linear trend of each time series was removed and the time series were normalized to a zero-mean and unit-variance. The time series were not further low-pass filtered because GCA using low lag orders operates on high-frequency deflections in time courses [33]. Unlike the kernel version of GCA used in Liao et al. [27], a linear multivariate GCA was applied to evaluate the relationship between the time series according to a generic multivariate autoregressive model [28], [33]:
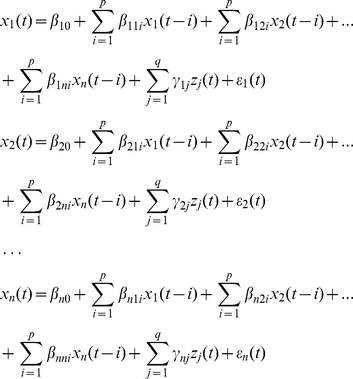
where *x_1_(t)*, …, *x_n_(t)* denotes *n* time series and *z_j_(t)* represents up to *q* exogenous covariates (6 head motion parameters and global mean signal) (*j = 1, …, q*). *p* denotes the autoregressive order and was set to 1 here to estimate the time-directed prediction between the BOLD time series across a lag of one TR (2000 ms) because an order of 1 can maximize the temporal resolution of the estimates of neural influence [29], [31], [33]. Granger causality coefficients (GCCs) are defined by *β* in this formula and denote the contributions of each lagged variable to the prediction of its respective target. *γ* corresponds to the covariate effect, and the prediction errors of individual models are denoted by *ε*. If a GCC *β_nm_* is significantly different from zero, then it is said that *x_m_* Granger causes *x_n_*. Two-tailed one-sample t-tests were performed for all of the possible 90×89 pair-wise GCCs across subjects. A false discovery rate (FDR) correction [41] was used to control the expected false discovery rate at 0.05 (corrected *P*<0.05). Using this resultant threshold, we converted the causality matrix into a binarized matrix (sparsity = 7.44%). The sparsity of a network is the ratio of the number of existing directed edges to the maximum possible directed edges in the network and whose element was 1 if there was significant Granger causality from one brain region to another and 0 otherwise. Thus, we constructed a population-based functional directed network by capturing the underlying common connectivity pattern of the brain (i.e., backbone), which controls the “false-positive” connections to be minimal in the network.

### Network analysis

#### Nodal degree

For a given node *i*, the out-degree was the number of outflow connections from node *i* to any other node in the network and quantified the driving ability of this node [42]:
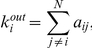
where *N* is the number of nodes and *a_ij_* denotes the directed connection from node *i* to node *j*.

In-degree was the number of inflow connections to a node from any other node in the network and quantified the receiving ability of this node:
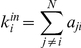
Out-In degree was the difference between out-degree and in-degree and measured the net outflow from a node:
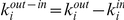



The nodes with the largest degree values were considered pivotal nodes (i.e., hubs) in the network. Specifically, we identified driving hubs in the functional directed network as those nodes with out-degree values of at least one standard deviation (SD) greater than the average out-degree of the network (i.e., 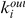
>mean+SD). Likewise, we identified driven hubs according to their in-degree (i.e., 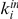
>mean+SD).

#### Small-world properties

The small-world model of undirected networks was originally proposed by Watts and Strogatz [43]. Small-world networks have highly local clustering (i.e., neighboring nodes are connected tightly) and short average paths (i.e., one node is only a few paths away from any other node in the network), thereby supporting the coexistence of segregation and integration. In this study, we investigated the small-world properties of directed brain networks. The directed clustering coefficient, *C_d_*, of a network was the average of the clustering coefficients of all nodes whereas the clustering coefficient, *C_i_*, of a node *i* was defined as the likelihood that the node's neighbors were connected with each other [44]:
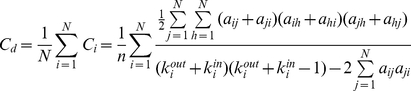
The directed clustering coefficient, *C_d_*, quantified the extent of local cliquishness or the local efficiency of information transfer of a network [43]–[45].

The path length from node *i* to node *j* was defined as the sum of the directed edge lengths along this path. The shortest path length, *L_ij_*, from node *i* to node *j* was the length of the path with the shortest length between the two nodes. The directed characteristic shortest path length, *L_d_*, of a network was measured using a “harmonic mean” length between pairs as proposed by Newman [46], which is the reciprocal of the average of the reciprocals:
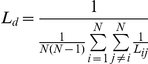
The directed characteristic shortest path length, *L_d_*, quantified the ability of a network to propagate information in parallel or the global efficiency (in terms of 1/*L_d_*) of a network.

The normalized directed clustering coefficient, 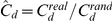, and the normalized directed characteristic path length, 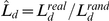, were also computed, where 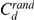 and 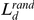 were the mean directed clustering coefficients and the directed characteristic path lengths of 100 matched random networks, respectively. These matched random networks were generated by preserving the same number of nodes, edges, out-degree and in-degree distribution as the real networks [47]. A real network is considered small-world if it meets the following criteria: 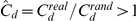 and 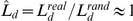 [43]. In other words, a small-world network has a much higher local efficiency than random networks but still approximately preserves the high global efficiency of the random networks. The small-world properties of the directed networks were calculated with the Brain Connectivity Toolbox [42] (http://www.brain-connectivity-toolbox.net/).

#### Modularity

Modularity is one of the most fundamental and intriguing properties of many biological networks [48]. To explore the intrinsic modular structure of the human brain directed network, we computed modularity according to Leicht and Newman's algorithm [49]. The modularity, *Q*, for a given partition, *p*, of the directed network was defined as:
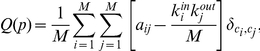
where *M* was the number of edges, 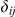 was the Kronecker delta symbol, and *c_i_* was the label of the module to which node *i* was assigned. The modularity index quantified the difference between the number of intra-module links of the actual network and the random network, in which the connections are linked at random. If one partition maximized *Q* over the possible divisions of the network, the maximum was considered as the best estimate of the true communities in the network. An explicit algorithm based on the spectral optimization of the modularity developed by Leicht and Newman [49] was used. Finally, we evaluated the significance of the modularity of the functional brain networks by comparing with the 100 node-, edge-, in-degree- and out-degree-matched random networks.

### Reproducibility of directed brain networks

#### 1) Threshold effects

Considering that the different thresholds have effects on the number of edges of the resulting brain networks and thereby influence the topological properties, we evaluated the topological stability of the brain functional networks over a wide sparsity ranging from 5% to 50%. Note that at the FDR threshold (sparsity = 7.44%), the directed network was a weakly connected (WC) graph in which it was possible to reach any node starting from any other node by traversing the edges in free directions (i.e., not necessarily in the direction that the edges pointed). When the sparsity increased to 13.07%, the network became a strongly connected (SC) graph in which it was possible to reach any node starting from any other node by traversing the edges in the directions that the edges pointed. Therefore, we also checked the nodal and modular properties at this sparsity of the SC threshold.

#### 2) Inter-subject variability

Another concern was that the inter-subject variability may dramatically influence the reliability of the group analysis of fMRI [50]. This concern was especially high for the GCA because it can be influenced by the inter-region and inter-subject variability of hemodynamic responses [51]–[55]. To test the reproducibility of our results across participants, we divided all 86 participants into two independent subgroups (43 subjects for each subgroup, age- and gender-matched) and calculated the split-half reliability. For each subgroup, the brain functional network were separately constructed and analyzed with the same criterion of the aforementioned whole-group analyses (sparsity = 7.44%). The results of the two independent subgroups were compared to evaluate the reproducibility.

## Results

### Directed functional connections of the human brain functional network

**Figure 2:**
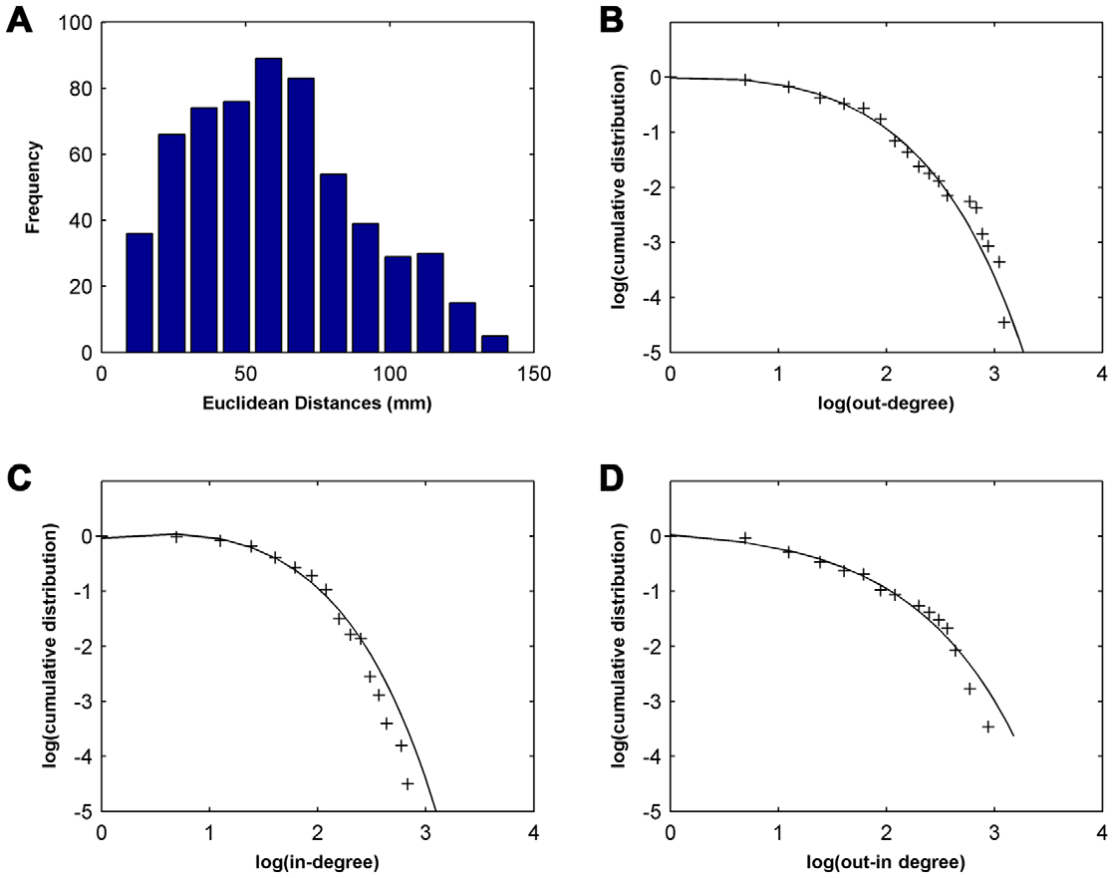
Distribution of anatomical distance and nodal degree. (**A**) Anatomical distance distribution. This figure shows numerous local-range connections and a few long-range connections. (**B**) Out-degree distribution. The exponentially truncated power-law (with an estimated exponent *α* = 1.29 and a cutoff degree *k_c_* = 4.26) fits in the log-log plot of the cumulative probability versus the out-degree of the brain functional directed network. (**C**) In-degree distribution. The exponentially truncated power-law (with an estimated exponent *α* = 1.57 and a cutoff degree *k_c_* = 3.13) fits in the log-log plot of the cumulative probability versus the in-degree of the brain functional directed network. (**D**) Out-In degree distribution. The exponentially truncated power-law (with an estimated exponent *α* = 1.03 and a cutoff degree *k_c_* = 6.12) fits in the log-log plot of the cumulative probability versus the out-in degree of the brain functional directed network.

At the statistical criterion (*P*<0.05, FDR corrected), 596 directed edges were significant in our studied population of young adults. One hundred and eight directed edges comprised 54 reciprocal connection pairs (one reciprocal connection pair between nodes *i* and *j* consisted of the two directed edges *a_ij_* and *a_ij_*), but the other 488 directed edges were one-way connections. We found 58.7% of the directed edges were intra-hemispheric connections, and 41.3% were inter-hemispheric connections. As demonstrated in Figure 2A, most of the significant directed edges had shorter anatomical distances (Euclidean distance <75 mm, 71.3%), but a few long-range (Euclidean distance >75 mm, 28.7%) directed edges were also observed in this brain network. Consistent with these results, several previous studies have also demonstrated many local and few long-range connections in the human brain [7], [8]. The short-range/local edges may be associated with the short fibers that constitute the local circuitry, but long-range edges may be associated with the commissural fibers (inter-hemispheric connections) and long intra-hemispheric association fibers [56]. Although the number of long-range connections is limited in the brain directed network, they might constitute shortcuts to ensure short mean path lengths of the whole network [4], [57]. Of note, there are some inter-hemispheric connections passing through long fibers, but they were defined as short Euclidean distances here. In the future studies, it would be important to employ fiber length acquired by diffusion tensor imaging to define the anatomical distances between brain regions. Table 1 includes the 20 most significant inter-regional directed connections (*P*≤1.10×10^−13^). Eleven of the 20 significant causal influences were present in homologous regions in a bilateral and symmetrical fashion.

**Table 1:** Twenty of the most significant directed connections.

Region (out)	Region (in)	Class	*P*
PUT.R	PUT.L	SIeH	9.18×10^−25^
PUT.L	PUT.R	SIeH	5.79×10^−22^
STG.R	STG.L	SIeH	6.19×10^−22^
ORBinf.L	SFGmed.L	IaH	1.16×10^−20^
IFGoperc.R	SMG.R	IaH	2.26×10^−17^
FFG.R	MOG.R	IaH	3.86×10^−17^
IFGoperc.R	IPL.R	IaH	6.61×10^−17^
PCL.L	PCL.R	SIeH	9.11×10^−17^
FFG.R	FFG.L	SIeH	2.49×10^−16^
CAL.R	CAL.L	SIeH	4.27×10^−16^
CAU.R	CAU.L	SIeH	1.07×10^−15^
HIP.R	HIP.L	SIeH	1.37×10^−15^
ACG.R	ACG.L	SIeH	3.56×10^−15^
SMG.R	SMG.L	SIeH	4.79×10^−15^
FFG.L	FFG.R	SIeH	5.39×10^−15^
IFGoperc.R	MFG.R	IaH	1.29×10^−14^
IFGoperc.L	IFGtriang.L	IaH	5.67×10^−14^
ORBinf.L	TPOsup.L	IaH	8.26×10^−14^
ACG.L	DCG.L	IaH	8.89×10−^14^
IFGoperc.L	IPL.L	IaH	1.10×10^−13^

List of the 20 directed edges (in a descending order of statistical significance) with the most significant Granger causality from Region (out) to Region (in). These edges were classified into intra-hemispheric (IaH, 9) and symmetrically inter-hemispheric (SIeH, 11) connections. *P* denotes the significance level of the Granger causality between the two regions. PUT, putamen; STG, superior temporal gyrus; ORBinf, orbital part of the inferior frontal gyrus; SFGmed, medial superior frontal gyrus; IFGoperc, opercular part of the inferior frontal gyrus; SMG, supramarginal gyrus; FFG, fusiform gyrus; MOG, middle occipital gyrus; IPL, inferior parietal lobule; PCL, paracentral lobule; CAL, calcarine fissure and surrounding cortex; CAU, caudate; HIP, hippocampus; ACG, anterior cingulate and paracingulate gyri; MFG, middle frontal gyrus; IFGtriang, triangular part of the inferior frontal gyrus; TPOsup, temporal pole (superior); DCG, middle cingulate and paracingulate gyri; L, left; R, right.

### Small-world brain functional directed networks

We calculated the directed clustering coefficient (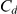) and directed characteristic path length (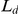) for both the functional directed network and the corresponding 100 random networks with the same number of nodes, edges, out-degree and in-degree distribution for the brain directed network. As expected, the directed network at the FDR corrected threshold (sparsity = 7.44%) demonstrated small-world architecture; it had an almost identical path length (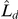 = 1.04) but was more locally clustered (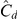 = 1.66) compared to the matched random networks. This result is compatible with previous directed network studies in animals and undirected network studies in humans (for reviews, see [58]–[61]). Liao et al. [27] found that the directed brain functional networks had significant but weak small-world properties. Our results showed that the directed brain functional networks exhibited prominent small-world properties, which obviously improved the previous results due to the methodological enhancements.

### Nodal degree and hub regions

We examined the nodal degree distribution of the directed network in the human brain. The brain network can be well fitted by an exponentially truncated power-law form, 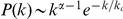, for the out-degree, in-degree and out-in degree (Figure 2). An exponentially truncated power-law degree distribution has been found in previous cortical anatomical networks [20], human brain structural networks [4], [6], [62] and functional networks [7]. When comparing networks with a scale-free (i.e., power-law) distribution, networks with a truncated power-law degree distribution are highly resilient to random errors and targeted attacks [7], [63]. This truncated power-law distribution indicates that the human brain network has some “core” regions but prevents the appearance of huge hubs with many connections.

**Figure 3:**
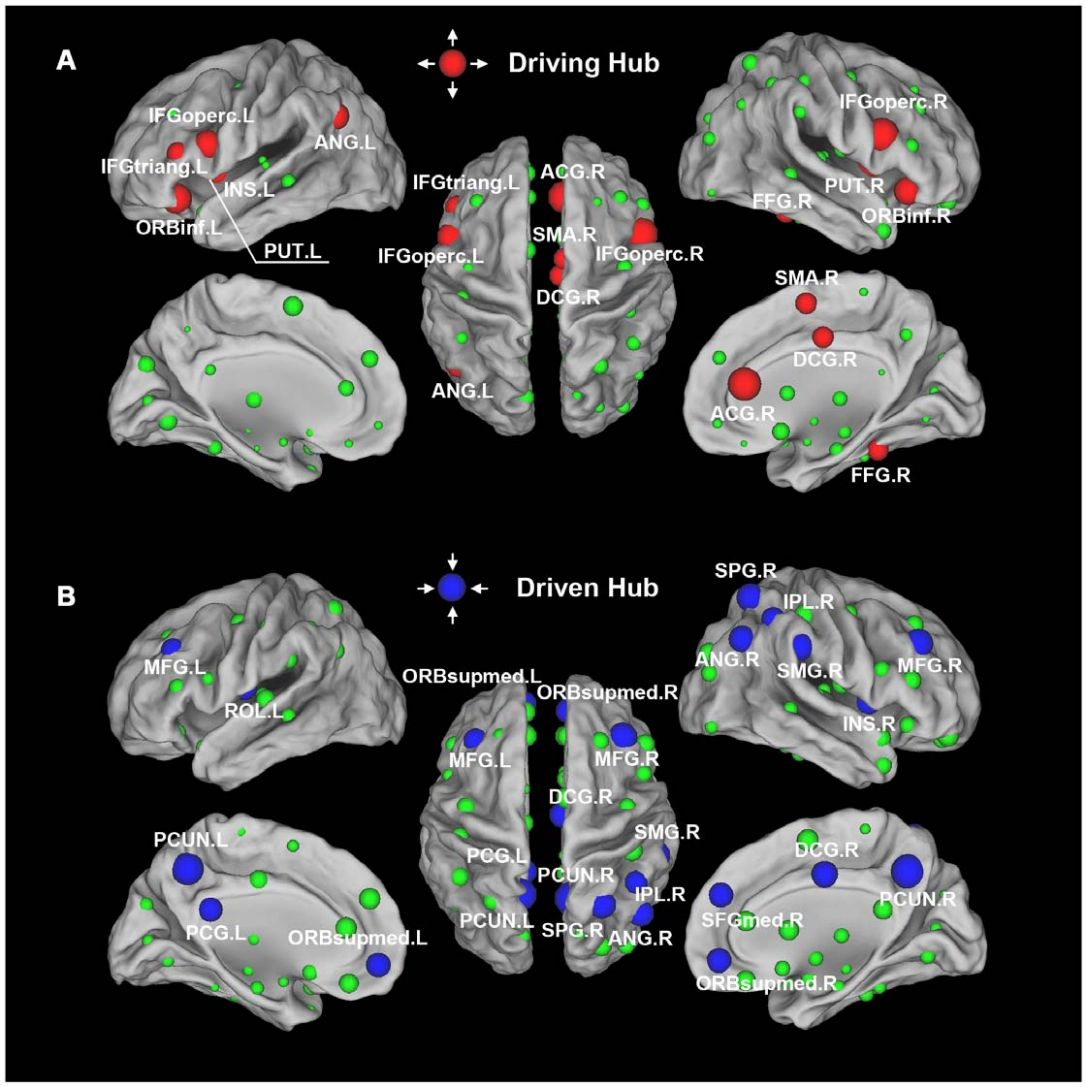
The driving and driven hubs in the human brain functional directed network. The surface visualization (using the Caret software [94]) of all 90 brain regions with node sizes indicating their relative out-degree 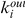 (**A**) or in-degree 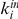 (**B**) values. (**A**) Regions with out-degree 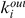
>mean+SD are considered driving hubs (red colors) or non-hubs (green colors) otherwise. (**B**) Regions with in-degree 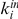
>mean+SD are considered driven hubs (blue colors) or non-hubs (green colors) otherwise. For the abbreviations of the regions, see Table S1.

To identify the hub regions in the human brain functional directed network, we examined the out-degree and in-degree of brain regions at the FDR threshold. Thirteen regions were identified as driving hubs, which are predominantly located in the attentional network [64]–[66], because of their large values of out-degree (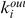
>mean+SD) (Figure 3A, Table 2). These driving hubs included 6 regions of the heteromodal or unimodal association cortex [the bilateral opercular part of the inferior frontal gyrus (IFGoperc), the left triangular part of the inferior frontal gyrus (IFGtriang), the right fusiform gyrus (FFG), the supplementary motor area (SMA) and the left angular gyrus (ANG)], 5 regions of the paralimbic cortex [the bilateral orbital part of the inferior frontal gyrus (ORBinf), the right anterior cingulate and paracingulate gyri (ACG), the middle cingulate and paracingulate gyri (DCG) and the left insula (INS)] and 2 regions of the subcortical cortex [the bilateral putamen (PUT)]. Fifteen regions were identified as driven hubs predominantly located in the default mode network (DMN) [67]–[69] because of their large values of in-degree (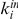
>mean+SD) (Figure 3B, Table 3). These driven hubs included 10 regions of the heteromodal or unimodal association cortex [the bilateral precuneus (PCUN), middle frontal gyrus (MFG), right superior parietal gyrus (SPG), inferior parietal lobule (IPL), medial superior frontal gyrus (SFGmed), supramarginal gyrus (SMG), ANG and left Rolandic operculum (ROL)] and 5 regions of the paralimbic cortex [the bilateral medial orbital part of the superior frontal gyrus (ORBsupmed), right DCG, INS and the left posterior cingulate gyrus (PCG)]. Most (2/3) of these driven regions located in the heteromodal or unimodal association cortex are consistent with previous undirected functional connectivity studies [3], [7]. Ten of the 13 driving hubs showed high net out-flow (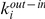
>mean+SD), whereas 10 of the 15 driven hubs showed high net in-flow (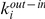
<mean−SD).

**Table 2:** Driving hub regions in the functional directed network of the human brain.

Driving hub regions	Class	Out-degree
IFGoperc.R	Association	22
ACG.R	Paralimbic	21
PUT.R	Subcortical	21
ORBinf.L	Paralimbic	19
IFGoperc.L	Association	18
ORBinf.R	Paralimbic	17
INS.L	Paralimbic	17
ANG.L	Association	17
PUT.L	Subcortical	16
IFGtriang.L	Association	13
SMA.R	Association	12
DCG.R	Paralimbic	12
FFG.R	Association	12

The driving hub regions (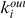
>mean+SD) in the human functional directed network are listed in a descending order of their out-degree 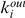. The regions are classified as primary, association, limbic, paralimbic and subcortical as described previously in [89]. IFGoperc, opercular part of the inferior frontal gyrus; ACG, anterior cingulate and paracingulate gyri; PUT, putamen; ORBinf, orbital part of the inferior frontal gyrus; INS, insula; ANG, angular gyrus; IFGtriang, triangular part of the inferior frontal gyrus; SMA, supplementary motor area; DCG, middle cingulate and paracingulate gyri; FFG, fusiform gyrus; L, left; R, right.

**Table 3:** Driven hub regions in the functional directed network of the human brain.

Driven hub regions	Class	In-degree
PCUN.R	Association	17
PCUN.L	Association	16
MFG.R	Association	14
SPG.R	Association	13
SMG.R	Association	13
DCG.R	Paralimbic	12
ANG.R	Association	12
MFG.L	Association	11
SFGmed.R	Association	11
ROL.L	Association	11
PCG.L	Paralimbic	11
ORBsupmed.R	Paralimbic	11
INS.R	Paralimbic	11
ORBsupmed.L	Paralimbic	11
IPL.R	Association	10

The driven hub regions (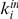
>mean+SD) in the human functional directed network are listed in a descending order of their in-degree 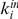. PCUN, precuneus; MFG, middle frontal gyrus; SPG, superior parietal gyrus; SMG, supramarginal gyrus; DCG, middle cingulate and paracingulate gyri; ANG, angular gyrus; SFGmed, medial superior frontal gyrus; ROL, Rolandic operculum; PCG, posterior cingulate gyrus; ORBsupmed, medial orbital part of the superior frontal gyrus; INS, insula; IPL, inferior parietal lobule; L, left; R, right.

### Modularity of the brain functional directed network

**Figure 4:**
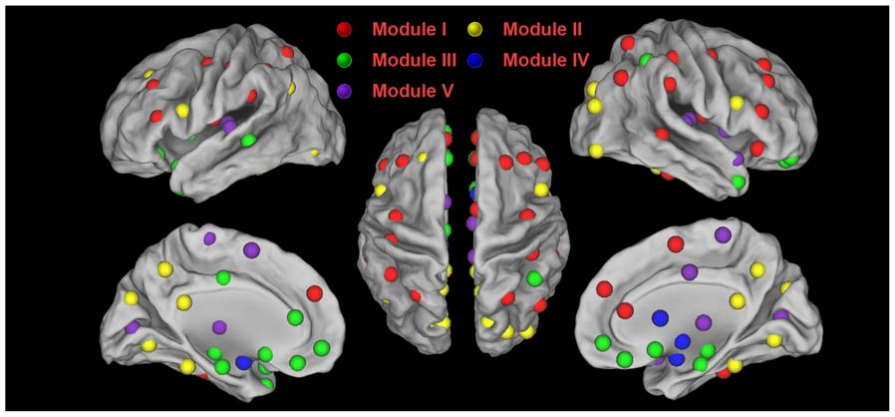
The modular architecture of the human brain functional directed network. All of the 90 brain regions are marked with different colored spheres (different colors represent distinct network modules) and further mapped onto the cortical surfaces using the Caret software [94].

We performed a modular detection process that did not take prior knowledge regarding the functionality of any brain regions into account. As a result, a maximum modularity (*Q_max_* = 0.32, Z-score = 10.80) was reached when the brain functional network was separated into 5 modules (I, II, III, IV, V in Figure 4 and Table 4). Module I was designated the “fronto-parietal” module. This module included 25 regions mainly from the frontal and parietal regions, such as the bilateral MFG, IFGtriang, SFGmed, SPG, precentral gyrus (PreCG), postcentral gyrus (PoCG), Rolandic operculum (ROL), SMG, inferior temporal gyrus (ITG), right superior frontal gyrus (SFGdor), ORBinf, SMA, ACG, ANG, middle temporal gyrus (MTG) and the left IPL. Module II was designated the “visual” module. This module included 20 regions mainly from the visual cortex, such as the bilateral superior occipital gyrus (SOG), middle occipital gyrus (MOG), inferior occipital gyrus (IOG), cuneus (CUN), lingual gyrus (LING), FFG, PCUN, PCG, IFGoperc, left SFGdor and ANG. Module III was designated the “paralimbic/limbic” module. This module included 23 regions mainly from the paralimbic and limbic cortex, such as the bilateral orbital part of the superior frontal gyrus (ORBsup), orbital part of the middle frontal gyrus (ORBmid), ORBsupmed, olfactory cortex (OLF), gyrus rectus (REC), temporal pole (middle) (TPOmid), hippocampus (HIP), parahippocampus gyrus (PHG), left ORBinf, temporal pole (superior) (TPOsup), MTG, INS, ACG, DCG and the right inferior parietal lobule (IPL). Module IV was designated the “subcortical” module. This module included 8 regions mainly from the subcortical cortex, such as the bilateral caudate (CAU), PUT, pallidum (PAL) and the amygdala (AMYG). Module V was designated the “primary” module. This module included 14 regions mainly from the auditory, visual and motor cortex, such as the bilateral Heschl's gyrus (HES), superior temporal gyrus (STG), calcarine fissure and the surrounding cortex (CAL), paracentral lobule (PCL), thalamus (THA), right INS, DCG, TPOsup and the left SMA.

**Table 4:** Modular architecture of the human brain functional directed network.

Module	Regions	Class	Module	Regions	Class
I	SFGdor.R	Association	III	ORBsup.L	Paralimbic
I	MFG.L	Association	III	ORBsup.R	Paralimbic
I	MFG.R	Association	III	ORBmid.L	Paralimbic
I	IFGtriang.L	Association	III	ORBmid.R	Paralimbic
I	IFGtriang.R	Association	III	ORBinf.L	Paralimbic
I	ROL.L	Association	III	ORBsupmed.L	Paralimbic
I	ROL.R	Association	III	ORBsupmed.R	Paralimbic
I	SMA.R	Association	III	REC.L	Paralimbic
I	SFGmed.L	Association	III	REC.R	Paralimbic
I	SFGmed.R	Association	III	INS.L	Paralimbic
I	SPG.L	Association	III	ACG.L	Paralimbic
I	SPG.R	Association	III	DCG.L	Paralimbic
I	IPL.L	Association	III	PHG.L	Paralimbic
I	SMG.L	Association	III	PHG.R	Paralimbic
I	SMG.R	Association	III	TPOsup.L	Paralimbic
I	ANG.R	Association	III	TPOmid.L	Paralimbic
I	MTG.R	Association	III	TPOmid.R	Paralimbic
I	ITG.L	Association	III	OLF.L	Limbic
I	ITG.R	Association	III	OLF.R	Limbic
I	PreCG.L	Primary	III	HIP.L	Limbic
I	PreCG.R	Primary	III	HIP.R	Limbic
I	PoCG.L	Primary	III	IPL.R	Association
I	PoCG.R	Primary	III	MTG.L	Association
I	ORBinf.R	Paralimbic	IV	CAU.L	Subcortical
I	ACG.R	Paralimbic	IV	CAU.R	Subcortical
II	SFGdor.L	Association	IV	PUT.L	Subcortical
II	IFGoperc.L	Association	IV	PUT.R	Subcortical
II	IFGoperc.R	Association	IV	PAL.L	Subcortical
II	CUN.L	Association	IV	PAL.R	Subcortical
II	CUN.R	Association	IV	AMYG.L	Limbic
II	LING.L	Association	IV	AMYG.R	Limbic
II	LING.R	Association	V	CAL.L	Primary
II	SOG.L	Association	V	CAL.R	Primary
II	SOG.R	Association	V	HES.L	Primary
II	MOG.L	Association	V	HES.R	Primary
II	MOG.R	Association	V	SMA.L	Association
II	IOG.L	Association	V	PCL.L	Association
II	IOG.R	Association	V	PCL.R	Association
II	FFG.L	Association	V	STG.L	Association
II	FFG.R	Association	V	STG.R	Association
II	ANG.L	Association	V	INS.R	Paralimbic
II	PCUN.L	Association	V	DCG.R	Paralimbic
II	PCUN.R	Association	V	TPOsup.R	Paralimbic
II	PCG.L	Paralimbic	V	THA.L	Subcortical
II	PCG.R	Paralimbic	V	THA.R	Subcortical

The modular architecture of the human brain functional directed network was detected using an explicit algorithm based on the spectral optimization of the modularity in directed networks developed by Leicht and Newman [49]. L, left; R, right; for the abbreviations of the regions, see Table S1.

### Reproducibility of our findings

#### 1) Threshold effects

**Figure 5:**
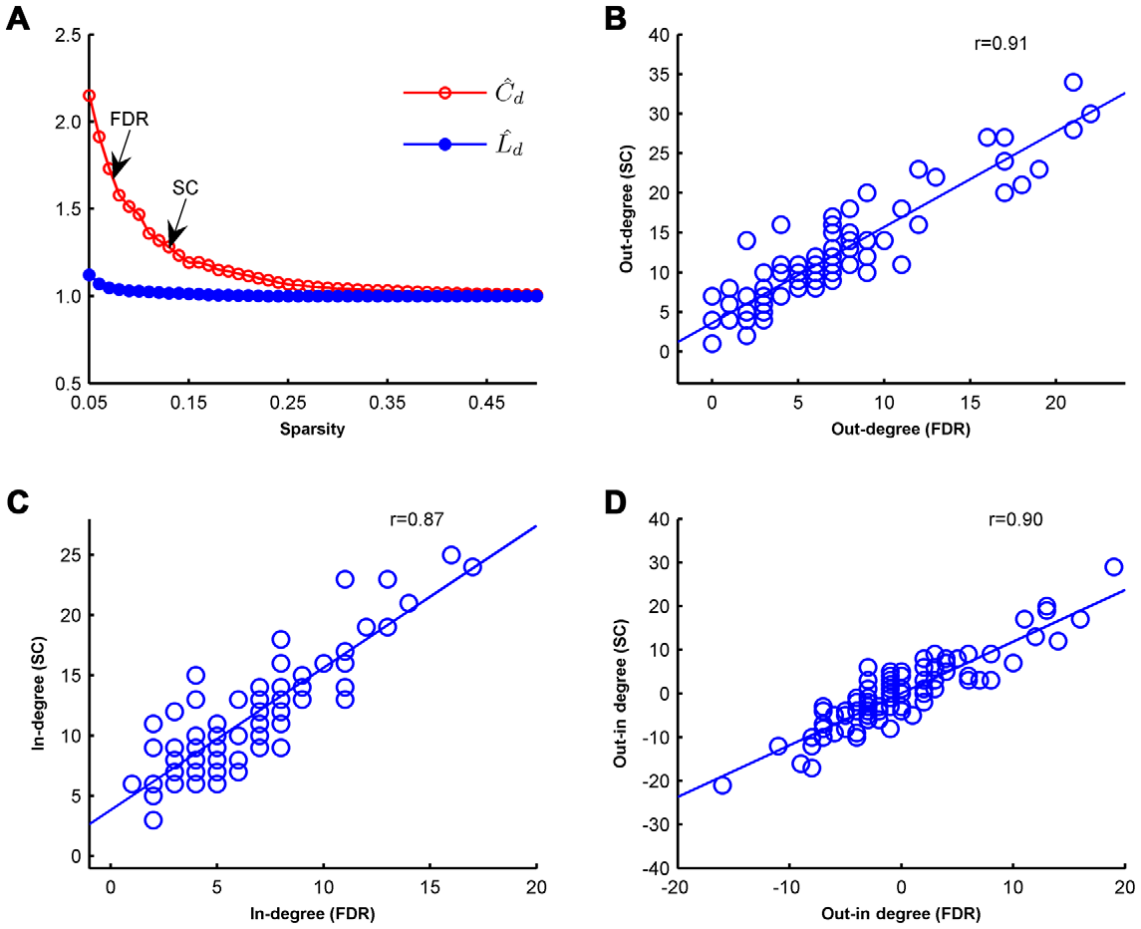
Sparsity-independent stability of the human brain functional directed network properties. (**A**) The directed networks demonstrated small-world architectures over a wide range of sparsity. For the FDR threshold (sparsity = 7.44%): 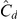 = 1.66, 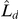 = 1.04; for the strongly connected (SC) threshold (sparsity = 13.07%): 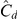 = 1.28, 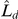 = 1.02. (**B–D**) The directed networks at these two sparsities (FDR threshold: 7.44% and SC threshold: 13.07%) showed high consistency on the nodal out-degree (*r* = 0.91, *P* = 4×10^−36^) (**B**), in-degree (*r* = 0.87, *P* = 4×10^−29^) (**C**) and out-in degree (*r* = 0.90, *P* = 3×10^−33^) (**D**).

**Figure 6:**
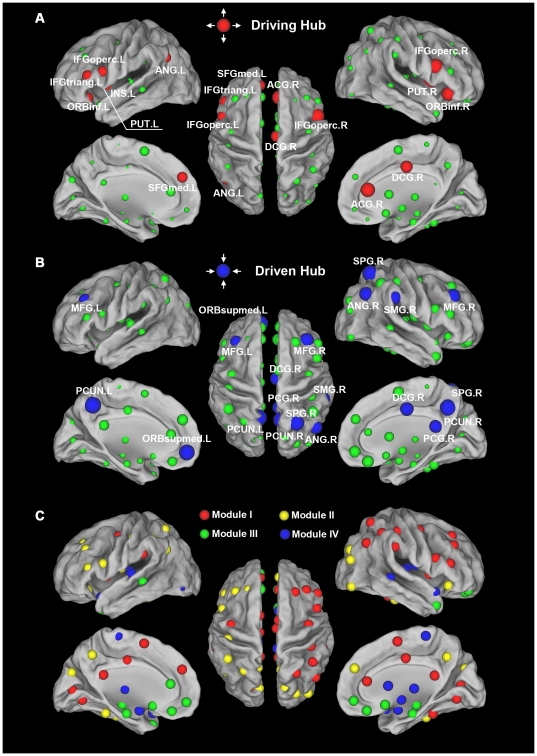
Hub distribution and modular architecture of the human brain functional directed network at strongly connected (SC) threshold. (**A**) Regions with out-degree 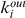
>mean+SD are considered driving hubs (red colors) or non-hubs (green colors) otherwise. (**B**) Regions with in-degree 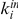
>mean+SD are considered driven hubs (blue colors) or non-hubs (green colors) otherwise. (**C**) All of the 90 brain regions are marked with different colored spheres (different colors represent distinct network modules) and further mapped onto the cortical surfaces using the Caret software [94]. For the abbreviations of the regions, see Table S1.

We evaluated the topological stability over a wide sparsity ranging from 5% to 50% for the whole group, and the directed network demonstrated small-world architectures (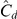
>1, 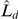
∼1) over this wide range of sparsity compared to the matched random networks (Figure 5A). As the sparsity increased to make the network a strongly-connected directed graph (SC threshold: sparsity = 13.07%), the normalized clustering coefficient of the brain network dropped but was still larger than 1 (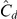 = 1.28); however, the normalized characteristic path lengths remained similar to 1 (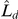 = 1.02). The directed networks at these two sparsities (SC threshold: 13.07% and FDR threshold: 7.44%) showed high consistency on the nodal out-degree (*r* = 0.91, *P* = 4×10^−36^), in-degree (*r* = 0.87, *P* = 4×10^−29^) and out-in degree (*r* = 0.90, *P* = 3×10^−33^) (Figures 5B, 5C and 5D). Twelve driving hubs were found at the SC threshold of which 11 were confirmed at the FDR threshold (sparsity = 7.44%), while 9 of the 10 driven hubs at the SC threshold had been found in the previous FDR threshold step (Figures 6A, 6B and Table S2). For the modular architecture at the SC threshold, Module IV (the “subcortical” module) and Module V (the “primary” module) were merged together and reduced to 4 modules while maintaining a similar modular organization with the FDR threshold (Figure 6C, Table S3). These results suggested that the properties of the directed network were not very sensitive to the selection of the sparsity thresholds.

#### 2) Inter-subject variability

**Figure 7:**
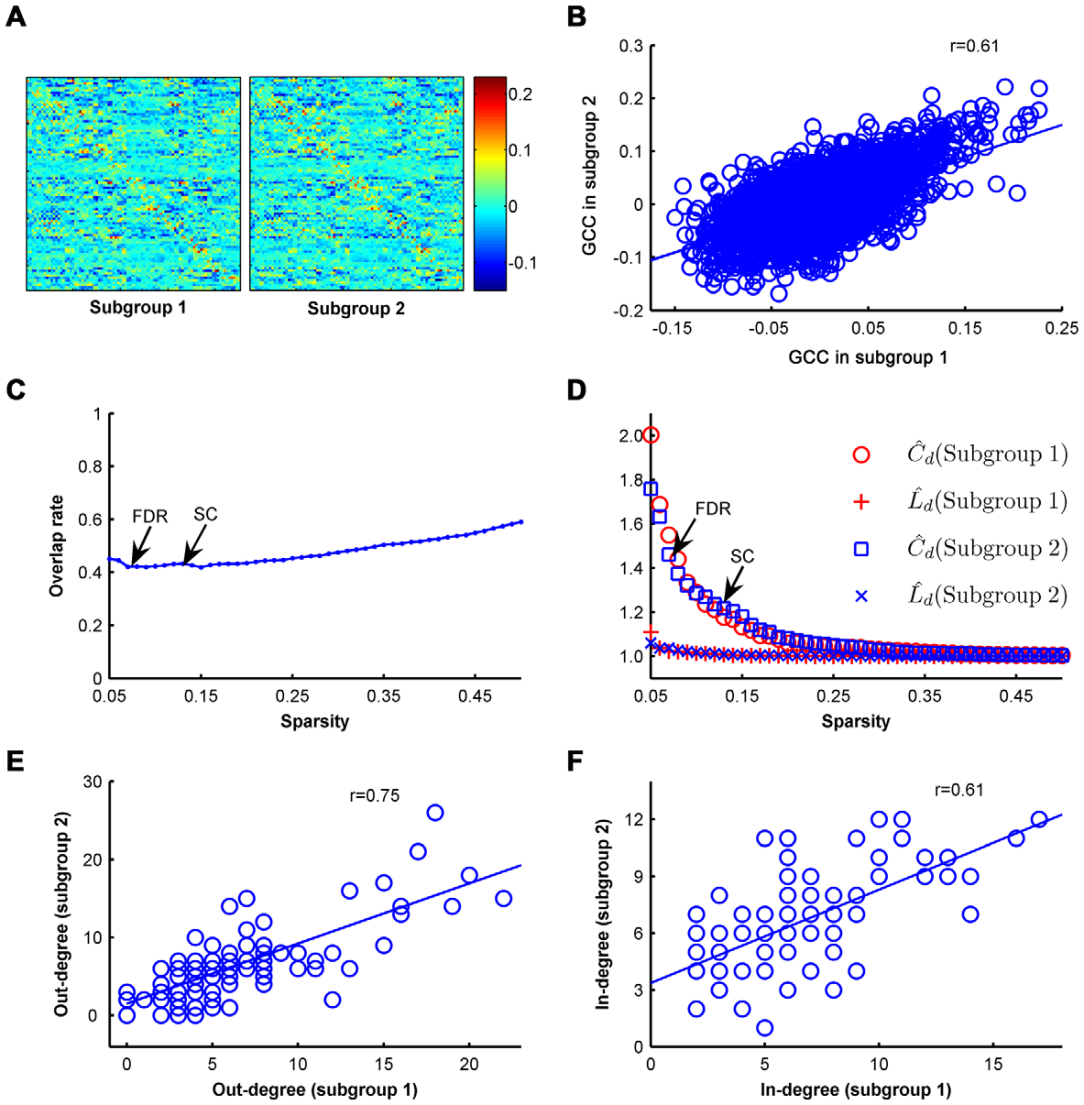
Split-half reproducibility of the human brain functional directed network properties. (**A**) The mean GCC patterns of the two subgroups were similar to each other and also similar to that in the aforementioned whole-group (Figure 1). (**B**) Significant correlation (*r* = 0.69) in the mean GCC between the two subgroups. (**C**) The overlap rate of the directed edges between the two subgroups. Overlap rate was defined as the percentage of edges in subgroup 1 can be replicated in subgroup 2 at a given sparsity. (**D**) For the two subgroups, the directed networks demonstrated small-world architectures over a wide range of sparsity. (**E**) The directed networks of the two subgroups showed high consistency on the nodal out-degree (*r* = 0.75, *P* = 3×10^−17^). (**F**) The directed networks of the two subgroups showed high consistency on the nodal in-degree (*r* = 0.61, *P* = 1×10^−10^).

We also calculated the split-half reliability by dividing all 86 participants into two independent subgroups (43 subjects for each subgroup, age- and gender-matched) to test the robustness of the construction of the brain functional directed network. Visual examination indicated that the GCC patterns were similar between the two datasets (Figure 7A) and in the aforementioned whole group (Figure 1). Further statistical analyses revealed a significant correlation (*r* = 0.69, *P* = 0.00) (Figure 7B) in the mean GCC between the two subgroups. The two subgroups showed a high overlap across a long range of sparsity (Figure 7C). Both of the directed networks of the two subgroups showed high small-world properties at the same threshold (sparsity = 7.44%) as the whole group (Figure 7D). The clustering coefficients of the brain networks for the two subgroups were approximately one and a half times greater than the comparable random networks (subgroup 1: 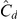 = 1.47; subgroup 2: 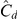 = 1.40), whereas the characteristic path length was approximately equivalent to the random networks (subgroup 1: 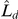 = 1.02; subgroup 2: 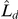 = 1.04). The directed networks of the two subgroups also showed high consistency in the nodal out-degree (*r* = 0.75, *P* = 3×10^−17^), in-degree (*r* = 0.61, *P* = 1×10^−10^) and out-in degree (*r* = 0.68, *P* = 3×10^−13^) (Figures 7E and 7F). Nine of the 13 driving hubs were confirmed in the two subgroups, and 8 of the 15 driven hubs were confirmed in the two subgroups (Figures S1A, S1B and Table S4). For the modular architecture in subgroup 1, Module I (the “fronto-parietal” module) and Module IV (the “subcortical” module) were merged together and reduced to 4 modules; however, the modular architecture in subgroup 2 retained 5 modules with small changes to the modular organization of the whole group (Figure S1C, Table S5 and Table S6). This split-half analysis demonstrated that the small-world topology, hub and modular structures showed high reproducibility between the two independent subgroups, suggesting that GCA might be a reliable approach to perform spontaneous causal influence analysis and construct directed network with R-fMRI.

## Discussion

In this study, we utilized GCA on R-fMRI data with a large sample of young healthy participants (n = 86) to construct spontaneous whole brain functional directed network of the human brain. We found that this directed network followed a small-world topology with significant modular structures that associated with 5 well known subsystems. Importantly, we identified driving hubs predominantly located in the attentional network as well as driven hubs predominantly located in the DMN. Furthermore, a split-half analysis demonstrated that the network properties showed high reproducibility between the two independent subgroups.

### GCA-based network construction and directed connections

Unlike other directed causal influence analysis techniques, such as structural equation modeling [70] and dynamic causal modeling [71], GCA incorporates information on temporal precedence and does not require *a priori* specification of a network model [29]–[31]. Therefore, GCA is suitable for the construction of directed network in the human brain. In the current study, we applied multivariate GCA to evaluate the relationship between the time series [28], [33], thus we can identify whether there was an intermediate node between two target nodes (i.e., to differentiate the Granger causality between X→Y and X→Z→Y). We further compared the 20 most significant edges with previous studies to address the possible neurobiological meaning of the directed edges detected by GCA (Table 1). Eleven of the 20 significant causal influences were present in homologous regions in a bilateral and symmetrical fashion. This finding is consistent with previous undirected functional connectivity studies that have shown coherent spontaneous activities between symmetrically bilateral brain regions, such as the bilateral motor cortex [72], visual cortex [73], auditory cortex [74], amygdala [73], caudate [75] and the putamen [75], [76]. Specifically, as the two most significant edges, the bi-directional information flow pathway between the bilateral putamen (also identified as driving hubs) can provide efficient information exchange to support the important roles of putamen that are involved in motoric and high-level cognitive functions [77]–[80]. In addition to the inter-hemispheric edges, we also observed significant intra-hemispheric causal edges mainly from the IFG (IFGoperc, ORBinf) to the SFG, MFG, IPL, TPOsup and SMG. Consistent with the current findings, a previous GCA study found that the inferior frontal cortex exerts significant causal influence on the dorso-lateral prefrontal cortex, posterior parietal cortex, posterior cingulate cortex and the temporo-parietal junction in resting-state [24]. The IFG is a critical region in response inhibition [81], [82] and plays a key role in switching between the central-executive and the DMN [24]. Therefore, the IFG might need to exert a causal influence on these distributed brain regions to maintain its important driving hub role.

### Small-world directed brain functional network in humans

Recent studies have demonstrated small-world topology in large-scale structural brain networks and functional undirected brain networks in humans (for reviews, see [58]–[61]). Several studies in cats and monkeys have found that the anatomical directed networks of animals are small-world [18]–[20]. However, a very recent R-fMRI study [27] shows that the small-world property of human functional directed network is very weak because the normalized clustering coefficients (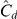) ranging from 1.02 to 1.08 for their given thresholds. Here, we showed that the directed brain functional network had a prominent small-world structure characterized by a higher normalized clustering coefficient (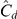 = 1.66). This result is compatible with previous AAL-based undirected brain functional networks in which the normalized clustering coefficients were around 2 [7], [8], [10], [32]. The discrepancy between our result and Liao et al.'s [27] could be due to the fact that they used a kernel version GCA that over-fits the data (further comments can be found in the following section). Our finding eliminates the doubt of the fundamental principle of small-world topology effects in the functional directed networks. The current study further supports that small-world topology is a key strategy in the organization of the complex brain network to make it an efficient neural architecture while maximizing the power of information processing [83], [84].

### Driving and driven neural hubs in the human brain

Most of the 13 driving hub regions (e.g., the inferior frontal gyrus [IFG] regions, SMA, INS, ACG and the FFG) are involved in the previously reported attentional or so-called “task-positive” network [64]–[66]. The task-positive network regions are routinely activated during goal-directed task performance and are anti-correlated with the DMN [64]–[66]. The brain regions in the task-positive network support an extrospectively-oriented mind state (i.e., to enter a mode of preparedness and alertness for possible changes) [65]. Previous GCA studies have found regions in this task-positive network, such as the IFG, which had the highest out-degree in our results, that show the highest out-degree and a large influence on other brain regions in salience tasks [24], [85] or in resting-state [24], [27]. Our findings suggest that the task-positive network could exert a large influence on other brain networks to maintain attention and readiness even in resting-state.

Fifteen regions were identified as driven hubs, and most of them (PCUN, PCG, MFG, SFGmed, ORBsupmed, IPL, ANG) are involved in the DMN [67]–[69] (Figure 3B, Table 3). Similar to our finding that the PCUN showed the highest inflow, Jiao et al. [25] found that the PCUN showed the strongest causal inflow among seven DMN regions using GCA. Liao et al. [27] found that the PCG/PCUN showed consistent high in-degree distribution. In addition, Deshpande et al. [26] also found that the PCG/PCUN acts as major hubs in bidirectional causal interactions. As the most important part of DMN, the PCUN and the adjacent PCG can be posited as a tonically-active region of the brain that may continuously gather information about the world around and within us [67]. As another important part of the DMN, the medial prefrontal cortex, which is heavily interconnected with limbic structures [86], [87], showed a high causal inflow here, which is suggested to be associated with an evaluation of the salience of the general information collected by PCUN and PCG [67]. Our findings further support the hypothesis that the DMN is broadly associated with the gathering and evaluating of information in resting-state [67].

Of note, Uddin et al. [66] found that the DMN exerts greater Granger influence on the task-positive network in resting-state, which seems inconsistent with our findings. There are several discrepancies between our study and theirs. First, they extracted the mean time series from big masks other than neural nodes (e.g., all of the voxels showed significant correlation with PCG for the DMN). Second, they did not take other brain regions into account. The driving hubs in the task-positive network may exert an influence on the executive network, and the DMN may gather information from the sensory network. Together, our findings suggest that, even in the resting-state without any attentional tasks, the DMN is receiving and evaluating general information continuously, while the task-positive network exerts influence on other brain networks, such as the executive network, to maintain a high readiness for incoming attentional events. These results give insight to the function of the task-positive network and the competition and/or cooperation between the task-positive network and the DMN.

### Modularity of the brain functional directed network

We found that the functional directed network of the human brain showed high modularity (*Q_max_* = 0.32, Z-score = 10.80), which is consistent with previous modularity studies in the human brain [9]–[13]. The current finding suggests that modular structure is a fundamental design principle of spontaneous brain functional directed networks which allows evolutionary or developmental adaptation of one functional module without risking loss of function in other modules [11], [88]. We identified five intrinsically cohesive modules corresponding to 5 well known subsystems: fronto-parietal, visual, paralimbic/limbic, subcortical and primary systems (Figure 4, Table 4). Most regions (37/45) in the first two modules (fronto-parietal, visual) were association cortex regions. Therefore, the modular architecture here is consistent with the cortex parcellation scheme of Mesulam [89]: association, limbic, paralimbic, subcortical and primary sensory areas. Our results are also consistent with the modular organization reported in several recent human brain networks studies. The visual module has been found in almost all of the human brain modularity studies [9], [11]–[13], [27]. The fronto-parietal module has been identified similarly as the fronto-cingulo-parietal module [11] or the attention module [9]. The limbic/paralimbic module and subcortical module have been found as a single module in the study of He et al. [9]. The discrepancies between the current study and previous functional studies could attribute to the directed modular architecture detection approach that can identify finer community structure by taking information flow directions into account [49]. The directed modular architecture might give insights for our understanding of the human brain functional connectome.

### Reproducibility

We constructed the human brain functional directed network using GCA that incorporates information on temporal precedence and does not require *a priori* specification of a network model [29]–[31]. Nevertheless, GCA in fMRI is still an open controversial issue because of the limitations imposed by the hemodynamic response and inter-subject variability [51]–[55]. To address these issues, we evaluated the sparsity-independent stability and split-half reproducibility of the GCA-based functional directed network of the human brain. We found that the intrinsic functional directed network showed high consistency in small-world properties, nodal degree distribution and modular architecture across sparsities (Figure 5 and Figure 6). Importantly, by dividing all 86 participants into two independent subgroups, we found that the Granger causality coefficients of the two subgroups showed a high consistency (Figure 7). The directed network for the two subgroups showed a high overlap rate, consistent driving/driven hub distribution and a similar modular architecture (Figure 7 and Figure S1). These results for the high sparsity-independent stability and high split-half reproducibility suggest that the GCA might be a reliable approach for the performance of a spontaneous causal influence analysis with R-fMRI.

### Discrepancies between the current study and previous studies of functional directed network

Most previous GCA studies on R-fMRI data have focused on a small number of regions rather than the whole brain functional directed network. Fox example, Sridharan et al. [24] focused on 8 regions within the DMN, the central-executive network and the salience network. Jiao et al. [25] focused on 7 regions within the DMN. Deshpande et al. [26] focused on 33 regions within 4 networks, such as the DMN, the hippocampal cortical memory network, dorsal attention network and the fronto-parietal control network. Of note, Liao et al. [27] performed a whole brain functional directed network study utilizing GCA on R-fMRI time series of 90 AAL regions. Our study is different from theirs in the following ways.

First, Liao et al. [27] used a kernel version of GCA that might over-fit the data and model too much noise. This may explain why they found a very weak small-world property as the normalized clustering coefficients (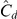) ranging from 1.02 to 1.08 for their given thresholds. Similar to Hamilton et al. [33], we used a linear multivariate GCA with intuitive interpretations for GCC, which could avoid the problems of fixing the degree of nonlinearity of the model and losing statistical power due to introducing more features with nonlinearity encountered in nonlinear generalization of GCA [33], [34]. We showed a much higher normalized clustering coefficient (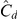 = 1.66), which is compatible with previous brain network studies [7], [8], [10], [32]. Second, Liao et al. [27] constructed directed network for each participant and analyzed the individual network properties. Notably, GCA may yield spurious connections (i.e., false positives) in the worst case scenario if the hemodynamic delay opposes the neuronal delay, and therefore, the causality needs to be statistically inferred [26]. In the current study, we constructed a population-based functional directed network by capturing the underlying common connectivity pattern of the cerebral cortex (i.e., backbone) across young healthy adults to control the “false-positive” connections to be minimal (e.g., using conservative statistical criterion) in the network. Third, Liao et al. [27] calculated each participant's degree properties and used the averaged degree across participants to identify the hub regions. This procedure did not consider the variation of different participants, although they used a randomization procedure of the time series to assess the statistical significance. Fourth, there were different number of time points between our study (n = 230) and Liao et al.'s study (n = 500), which may also contribute to the discrepancies for small-world results between the two studies. Further work could be conducted to compare the effects of the scan length of R-fMRI on graph theoretical analysis of brain networks. Finally, with a larger cohort of participants (N = 86), we divided all 86 participants into two independent subgroups (43 subjects for each subgroup, age- and gender-matched) and constructed population-based functional directed networks for each subgroup to evaluate the inter-subject variability. This analysis was not performed in previous directed brain functional studies. Our results of a high split-half reproducibility suggest that GCA might be a reliable approach to analyze spontaneous causal influence in brain functional networks.

### Further considerations

Several issues need to be further addressed. First, it has been reported that the deconvolution of the hemodynamic effects in fMRI time series before GCA can minimize spurious interactions due to the hemodynamic variability between brain regions [17]. It would be a prominent issue to evaluate and deconvolute the hemodynamic response of distinct brain regions of R-fMRI in the future. Second, the cardiac and respiratory cycles may have effects when evaluating the causal influence between brain regions. It would be crucial to record cardiac and respiratory signals simultaneously with fMRI scanning and to reduce these effects in GCA. Third, how the anatomical connections constrain the functional directed networks remains unknown. It would be important to integrate the structural network and the functional directed network modalities in the future. Fourth, previous electroencephalography (EEG) studies have demonstrated that alpha activity (EEG signals with frequencies 8–12) is present with eyes closed and that the sources of this activity are located in occipital cortical regions and the thalamus [90]–[92]. The dependency between EEG and fMRI is not straightforward and the link between fMRI and neuronal activity is frequency-dependent in a complex fashion [93]. Therefore, it's important to perform studies of simultaneous recording of EEG/Magnetoencephalography and fMRI to validate the current results regarding the patterns of the information flow.

### Conclusion

Using a linear multivariate GCA on R-fMRI data, we constructed a population-based whole-brain functional directed network and demonstrated that this network followed a small-world topology with significant modular structures. Thus, our study showed the improvement on the small-world topology as compared to a previous study [27] showing weak small-world properties using a kernel version of GCA and individual network analysis. Importantly, we identified driving hubs predominantly located in the attentional network as well as driven hubs predominantly located in the DMN. The current study provides a directed network perspective and demonstrates a spontaneous information flow and causal influence between distinct brain regions, which provides insight into our understanding of the brain functional architecture. Further work could be conducted to examine how the spontaneous functional directed network organization of the human brain is altered during normal development and aging as well as in specific brain disorders.

## Supporting Information

Figure S1Hub distribution and modular architecture of the human brain functional directed network for the two subgroups. (**A**) Regions with out-degree 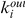
>mean+SD are considered driving hubs (red colors) or non-hubs (green colors) otherwise for subgroup 1 (Left) and subgroup 2 (Right). (**B**) Regions with in-degree 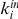
>mean+SD are considered driven hubs (blue colors) or non-hubs (green colors) otherwise for subgroup 1 (Left) and subgroup 2 (Right). (**C**) All of the 90 brain regions are marked with different colored spheres (different colors represent distinct network modules) and further mapped onto the cortical surfaces for subgroup 1 (Left) and subgroup 2 (Right). For the abbreviations of the regions, see Table S1.(TIF)

Table S1 Abbreviations for the regions in the AAL-atlas.
(DOC)

Table S2 Driving hub regions in the functional directed network of human brain at strongly connected (SC) threshold.
(DOC)

Table S3 Modular architecture of the brain functional directed network at strongly connected (SC) threshold.
(DOC)

Table S4 Driving and driven hub regions in the brain functional directed network for the two subgroups.
(DOC)

Table S5 Modular architecture of the brain functional directed network for subgroup 1.
(DOC)

Table S6 Modular architecture of the brain functional directed network for subgroup 2.
(DOC)
