# Supplementary figures and images for: Driving and Driven Architectures of Directed Small-World Human Brain Functional Networks
Source: PLoS One. 2011 Aug 12;6(8):e23460. doi: 10.1371/journal.pone.0023460 (PMC3155571; doi:10.1371/journal.pone.0023460)

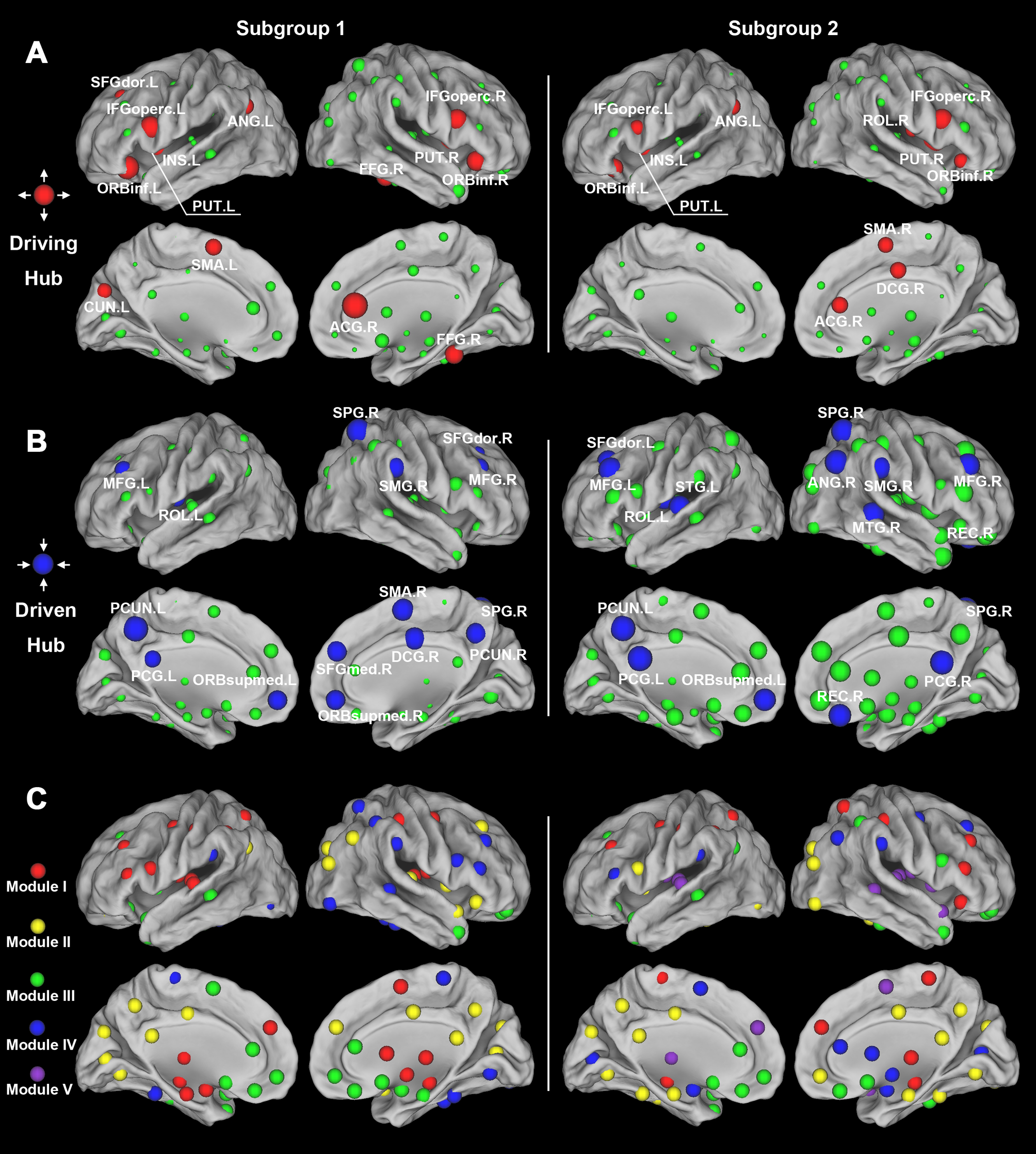

Supplement: Figure S1 — Hub distribution and modular architecture of the human brain functional directed network for the two subgroups. (A) Regions with out-degree >mean+SD are considered driving hubs (red colors) or non-hubs (green colors) otherwise for subgroup 1 (Left) and subgroup 2 (Right). (B) Regions with in-degree >mean+SD are considered driven hubs (blue colors) or non-hubs (green colors) otherwise for subgroup 1 (Left) and subgroup 2 (Right). (C) All of the 90 brain regions are marked with different colored spheres (different colors represent distinct network modules) and further mapped onto the cortical surfaces for subgroup 1 (Left) and subgroup 2 (Right). For the abbreviations of the regions, see Table S1. (TIF) [file pone.0023460.s001.tif]
