# Supplementary material for: Driving and Driven Architectures of Directed Small-World Human Brain Functional Networks
Source: PLoS One. 2011 Aug 12;6(8):e23460. doi: 10.1371/journal.pone.0023460 (PMC3155571; doi:10.1371/journal.pone.0023460)
Supplement: Table S1 — Abbreviations for the regions in the AAL-atlas. (DOC) [file pone.0023460.s002.doc]

**Table S1.** Abbreviations for the regions in the AAL-atlas.

| **Cortical regions** | **Abbreviations** |
| --- | --- |
| Precental gyrus | PreCG |
| Superior frontal gyrus, dorsolateral | SFGdor |
| Superior frontal gyrus, orbital part | ORBsup |
| Middle frontal gyrus | MFG |
| Middle frontal gyrus, orbital part | ORBmid |
| Inferior frontal gyrus, opercular part | IFGoperc |
| Inferior frontal gyrus, triangular part | IFGtriang |
| Inferior frontal gyrus, orbital part | ORBinf |
| Rolandic operculum | ROL |
| Supplementary motor area | SMA |
| Olfactory cortex | OLF |
| Superior frontal gyrus, medial | SFGmed |
| Superior frontal gyrus, medial orbital | ORBsupmed |
| Gyrus rectus | REC |
| Insula | INS |
| Anterior cingulate and paracingulate gyri | ACG |
| Middle cingulate and paracingulate gyri | DCG |
| Posterior cingulate gyrus | PCG |
| Hippocampus | HIP |
| Parahippocampal gyrus | PHG |
| Amygdala | AMYG |
| Calcarine fissure and surrounding cortex | CAL |
| Cuneus | CUN |
| Lingual gyrus | LING |
| Superior occipital gyrus | SOG |
| Middle occipital gyrus | MOG |
| Inferior occipital gyrus | IOG |
| Fusiform gyrus | FFG |
| Postcentral gyrus | PoCG |
| Superior parietal gyrus | SPG |
| Inferior parietal, but supramarginal and angular gyri | IPL |
| Supramarginal gyrus | SMG |
| Angular gyrus | ANG |
| Precuneus | PCUN |
| Paracentral lobule | PCL |
| Caudate nucleus | CAU |
| Lenticular nucleus, putamen | PUT |
| Lenticular nucleus, pallidum | PAL |
| Thalamus | THA |
| Heschl gyrus | HES |
| Superior temporal gyrus | STG |
| Temporal pole: superior temporal gyrus | TPOsup |
| Middle temporal gyrus | MTG |
| Temporal pole: middle temporal gyrus | TPOmid |
| Inferior temporal gyrus | ITG |
