# Supplementary material for: Driving and Driven Architectures of Directed Small-World Human Brain Functional Networks
Source: PLoS One. 2011 Aug 12;6(8):e23460. doi: 10.1371/journal.pone.0023460 (PMC3155571; doi:10.1371/journal.pone.0023460)
Supplement: Table S2 — Driving hub regions in the functional directed network of human brain at strongly connected (SC) threshold. (DOC) [file pone.0023460.s003.doc]

**Table S2. Driving hub regions in the functional directed network of human brain at strongly connected (SC) threshold.**

| Driving hub regions | Class | Out-degree |  | Driven hub regions | Class | In-degree |
| --- | --- | --- | --- | --- | --- | --- |
| PUT.R | Subcortical | 34 |  | PCUN.L | Association | 25 |
| IFGoperc.R | Association | 30 |  | PCUN.R | Association | 24 |
| ACG.R | Paralimbic | 28 |  | SPG.R | Association | 23 |
| ORBinf.R | Paralimbic | 27 |  | ORBsupmed.L | Paralimbic | 23 |
| PUT.L | Subcortical | 27 |  | MFG.R | Association | 21 |
| INS.L | Paralimbic | 24 |  | DCG.R | Paralimbic | 19 |
| ORBinf.L | Paralimbic | 23 |  | SMG.R | Association | 19 |
| DCG.R | Paralimbic | 23 |  | ANG.R | Association | 19 |
| IFGtriang.L | Association | 22 |  | PCG.R | Paralimbic | 18 |
| IFGoperc.L | Association | 21 |  | MFG.L | Association | 17 |
| SFGmed.L | Association | 20 |  |  |  |  |
| ANG.L | Association | 20 |  |  |  |  |

The driving hub regions ($k_{i}^{out}$ > mean + SD) and driven hub regions ($k_{i}^{in}$ > mean + SD) in the functional directed network at SC threshold were listed in a descending order of their out-degree $k_{i}^{out}$ or in-degree $k_{i}^{in}$. L, left; R, right; for the abbreviations of the regions, see Table S1.
