# Supplementary material for: Driving and Driven Architectures of Directed Small-World Human Brain Functional Networks
Source: PLoS One. 2011 Aug 12;6(8):e23460. doi: 10.1371/journal.pone.0023460 (PMC3155571; doi:10.1371/journal.pone.0023460)
Supplement: Table S3 — Modular architecture of the brain functional directed network at strongly connected (SC) threshold. (DOC) [file pone.0023460.s004.doc]

**Table S3. Modular architecture of the brain functional directed network at strongly connected (SC) threshold.**

| Module | Regions | Class |  | Module | Regions | Class |
| --- | --- | --- | --- | --- | --- | --- |
| I | SFGdor.R | Association |  | II | PCUN.R | Association |
| I | MFG.R | Association |  | II | ITG.L | Association |
| I | IFGoperc.R | Association |  | II | ITG.R | Association |
| I | IFGtriang.R | Association |  | II | ORBinf.L | Paralimbic |
| I | SMA.L | Association |  | II | ORBinf.R | Paralimbic |
| I | SMA.R | Association |  | II | PoCG.L | Primary |
| I | SFGmed.L | Association |  | III | ORBsup.L | Paralimbic |
| I | LING.L | Association |  | III | ORBsup.R | Paralimbic |
| I | LING.R | Association |  | III | ORBmid.L | Paralimbic |
| I | SOG.L | Association |  | III | ORBmid.R | Paralimbic |
| I | FFG.R | Association |  | III | ORBsupmed.L | Paralimbic |
| I | SPG.R | Association |  | III | ORBsupmed.R | Paralimbic |
| I | IPL.R | Association |  | III | REC.L | Paralimbic |
| I | SMG.L | Association |  | III | REC.R | Paralimbic |
| I | SMG.R | Association |  | III | ACG.L | Paralimbic |
| I | ANG.R | Association |  | III | PHG.L | Paralimbic |
| I | INS.L | Paralimbic |  | III | PHG.R | Paralimbic |
| I | INS.R | Paralimbic |  | III | TPOmid.L | Paralimbic |
| I | ACG.R | Paralimbic |  | III | TPOmid.R | Paralimbic |
| I | DCG.L | Paralimbic |  | III | OLF.L | Limbic |
| I | DCG.R | Paralimbic |  | III | OLF.R | Limbic |
| I | PCG.L | Paralimbic |  | III | HIP.L | Limbic |
| I | PCG.R | Paralimbic |  | III | HIP.R | Limbic |
| I | PreCG.L | Primary |  | III | MTG.L | Association |
| I | PreCG.R | Primary |  | IV | CAU.L | Subcortical |
| I | CAL.L | Primary |  | IV | CAU.R | Subcortical |
| I | CAL.R | Primary |  | IV | PUT.L | Subcortical |
| I | PoCG.R | Primary |  | IV | PUT.R | Subcortical |
| II | SFGdor.L | Association |  | IV | PAL.L | Subcortical |
| II | MFG.L | Association |  | IV | PAL.R | Subcortical |
| II | IFGoperc.L | Association |  | IV | THA.L | Subcortical |
| II | IFGtriang.L | Association |  | IV | THA.R | Subcortical |
| II | ROL.L | Association |  | IV | HES.L | Primary |
| II | SFGmed.R | Association |  | IV | HES.R | Primary |
| II | CUN.L | Association |  | IV | ROL.R | Association |
| II | CUN.R | Association |  | IV | IOG.L | Association |
| II | SOG.R | Association |  | IV | PCL.L | Association |
| II | MOG.L | Association |  | IV | PCL.R | Association |
| II | MOG.R | Association |  | IV | STG.L | Association |
| II | IOG.R | Association |  | IV | STG.R | Association |
| II | FFG.L | Association |  | IV | MTG.R | Association |
| II | SPG.L | Association |  | IV | AMYG.L | Limbic |
| II | IPL.L | Association |  | IV | AMYG.R | Limbic |
| II | ANG.L | Association |  | IV | TPOsup.L | Paralimbic |
| II | PCUN.L | Association |  | IV | TPOsup.R | Paralimbic |

The modular architecture of the brain functional directed network at SC threshold was detected using an explicit algorithm based on spectral optimization of the modularity in directed networks developed by Leicht and Newman (2008). L, left; R, right; for the abbreviations of the regions, see Table S1.
