# Supplementary material for: Driving and Driven Architectures of Directed Small-World Human Brain Functional Networks
Source: PLoS One. 2011 Aug 12;6(8):e23460. doi: 10.1371/journal.pone.0023460 (PMC3155571; doi:10.1371/journal.pone.0023460)
Supplement: Table S4 — Driving and driven hub regions in the brain functional directed network for the two subgroups. (DOC) [file pone.0023460.s005.doc]

**Table S4.** Driving and driven hub regions in the brain functional directed network for the two subgroups.

|  | Driving hub regions | Class | Out-degree |  | Driven hub regions | Class | In-degree |
| --- | --- | --- | --- | --- | --- | --- | --- |
| *Subgroup 1* | |  |  |  |  |  |  |
|  | ACG.R | Paralimbic | 22 |  | PCUN.L | Association | 17 |
|  | ORBinf.L | Paralimbic | 20 |  | SPG.R | Association | 16 |
|  | IFGoperc.L | Association | 19 |  | DCG.R | Paralimbic | 14 |
|  | PUT.R | Subcortical | 18 |  | SMA.R | Association | 14 |
|  | IFGoperc.R | Association | 17 |  | SMG.R | Association | 13 |
|  | ORBinf.R | Paralimbic | 16 |  | SFGmed.R | Association | 13 |
|  | PUT.L | Subcortical | 16 |  | PCUN.R | Association | 13 |
|  | INS.L | Paralimbic | 15 |  | ORBsupmed.L | Paralimbic | 12 |
|  | FFG.R | Association | 15 |  | ORBsupmed.R | Paralimbic | 12 |
|  | SMA.L | Association | 13 |  | MFG.L | Association | 11 |
|  | ANG.L | Association | 13 |  | MFG.R | Association | 11 |
|  | SFGdor.L | Association | 12 |  | PCG.L | Paralimbic | 10 |
|  | CUN.L | Association | 12 |  | ROL.L | Association | 10 |
|  |  |  |  |  | SFGdor.R | Association | 10 |
| *Subgroup 2* | |  |  |  |  |  |  |
|  | PUT.R | Subcortical | 26 |  | PCUN.L | Association | 12 |
|  | IFGoperc.R | Association | 21 |  | MFG.L | Association | 12 |
|  | ORBinf.L | Paralimbic | 18 |  | PCG.L | Paralimbic | 12 |
|  | INS.L | Paralimbic | 17 |  | MFG.R | Association | 11 |
|  | ANG.L | Association | 16 |  | SPG.R | Association | 11 |
|  | ACG.R | Paralimbic | 15 |  | MTG.R | Association | 11 |
|  | DCG.R | Paralimbic | 15 |  | ANG.R | Association | 11 |
|  | IFGoperc.L | Association | 14 |  | PCG.R | Paralimbic | 11 |
|  | SMA.R | Association | 14 |  | ROL.L | Association | 10 |
|  | PUT.L | Subcortical | 14 |  | SMG.R | Association | 10 |
|  | ORBinf.R | Paralimbic | 13 |  | STG.L | Association | 10 |
|  | ROL.R | Association | 12 |  | REC.R | Paralimbic | 10 |
|  |  |  |  |  | SFGdor.L | Association | 10 |
|  |  |  |  |  | ORBsupmed.L | Paralimbic | 10 |

The driving hub regions ($k_{i}^{out}$ > mean + SD) and driven hub regions ($k_{i}^{in}$ > mean + SD) in the functional directed network for the two subgroups were listed in a descending order of their out-degree $k_{i}^{out}$ or in-degree $k_{i}^{in}$. L, left; R, right; for the abbreviations of the regions, see Table S1.
