# Supplementary material for: Driving and Driven Architectures of Directed Small-World Human Brain Functional Networks
Source: PLoS One. 2011 Aug 12;6(8):e23460. doi: 10.1371/journal.pone.0023460 (PMC3155571; doi:10.1371/journal.pone.0023460)
Supplement: Table S5 — Modular architecture of the brain functional directed network for subgroup 1. (DOC) [file pone.0023460.s006.doc]

**Table S5.** Modular architecture of the brain functional directed network for subgroup 1.

| Module | Regions | Class |  | Module | Regions | Class |
| --- | --- | --- | --- | --- | --- | --- |
| I | MFG.L | Association |  | II | INS.R | Paralimbic |
| I | IFGoperc.L | Association |  | II | DCG.L | Paralimbic |
| I | IFGtriang.L | Association |  | II | DCG.R | Paralimbic |
| I | ROL.L | Association |  | II | PCG.L | Paralimbic |
| I | ROL.R | Association |  | II | PCG.R | Paralimbic |
| I | SMA.R | Association |  | II | TPOsup.R | Paralimbic |
| I | SFGmed.L | Association |  | II | CAL.L | Primary |
| I | SPG.L | Association |  | II | CAL.R | Primary |
| I | IPL.L | Association |  | III | ORBsup.R | Paralimbic |
| I | STG.L | Association |  | III | ORBmid.R | Paralimbic |
| I | CAU.L | Subcortical |  | III | ORBinf.L | Paralimbic |
| I | CAU.R | Subcortical |  | III | ORBsupmed.L | Paralimbic |
| I | PUT.L | Subcortical |  | III | ORBsupmed.R | Paralimbic |
| I | PUT.R | Subcortical |  | III | REC.L | Paralimbic |
| I | PAL.L | Subcortical |  | III | REC.R | Paralimbic |
| I | PAL.R | Subcortical |  | III | INS.L | Paralimbic |
| I | THA.L | Subcortical |  | III | ACG.L | Paralimbic |
| I | THA.R | Subcortical |  | III | ACG.R | Paralimbic |
| I | PreCG.L | Primary |  | III | PHG.R | Paralimbic |
| I | PreCG.R | Primary |  | III | TPOsup.L | Paralimbic |
| I | PoCG.L | Primary |  | III | TPOmid.L | Paralimbic |
| I | PoCG.R | Primary |  | III | TPOmid.R | Paralimbic |
| I | HES.L | Primary |  | III | OLF.L | Limbic |
| I | HES.R | Primary |  | III | OLF.R | Limbic |
| I | HIP.L | Limbic |  | III | AMYG.R | Limbic |
| I | HIP.R | Limbic |  | III | SFGdor.L | Association |
| I | AMYG.L | Limbic |  | III | SMA.L | Association |
| I | ORBmid.L | Paralimbic |  | III | MTG.L | Association |
| I | PHG.L | Paralimbic |  | III | ITG.L | Association |
| II | SFGdor.R | Association |  | IV | MFG.R | Association |
| II | SFGmed.R | Association |  | IV | IFGoperc.R | Association |
| II | CUN.L | Association |  | IV | IFGtriang.R | Association |
| II | CUN.R | Association |  | IV | LING.R | Association |
| II | LING.L | Association |  | IV | IOG.L | Association |
| II | SOG.L | Association |  | IV | IOG.R | Association |
| II | SOG.R | Association |  | IV | FFG.L | Association |
| II | MOG.L | Association |  | IV | FFG.R | Association |
| II | MOG.R | Association |  | IV | SPG.R | Association |
| II | ANG.L | Association |  | IV | IPL.R | Association |
| II | ANG.R | Association |  | IV | SMG.L | Association |
| II | PCUN.L | Association |  | IV | SMG.R | Association |
| II | PCUN.R | Association |  | IV | PCL.L | Association |
| II | STG.R | Association |  | IV | PCL.R | Association |
| II | ORBsup.L | Paralimbic |  | IV | MTG.R | Association |
| II | ORBinf.R | Paralimbic |  | IV | ITG.R | Association |

The modular architecture of the brain functional directed network for subgroup 1 was detected using an explicit algorithm based on spectral optimization of the modularity in directed networks developed by Leicht and Newman (2008). L, left; R, right; for the abbreviations of the regions, see Table S1.
